# Massive Sampling Strategy for Antibody–Antigen Targets in CAPRI Round 55 With MassiveFold

**DOI:** 10.1002/prot.26802

**Published:** 2025-01-27

**Authors:** Nessim Raouraoua, Marc F. Lensink, Guillaume Brysbaert

**Affiliations:** ^1^ Univ. Lille, UMR 8576 ‐ UGSF ‐ Unité de Glycobiologie Structurale et Fonctionnelle Lille France

**Keywords:** AlphaFold, antibody binding, CAPRI, MassiveFold, protein structure prediction, structural bioinformatics

## Abstract

Massive sampling with AlphaFold2 improves protein–protein complex predictions. This has been shown during the last CASP15‐CAPRI blind prediction round by the AFsample tool. However, more difficult targets including antibody–antigen binding remain challenging. CAPRI Round 55 consisted of three antibody–antigen targets and one heterotrimer. We used our AlphaFold2‐based MassiveFold, running 6 prediction pools, each with their own set of parameters, to produce in total more than 6000 predictions per target. We show here that massive sampling categorically produces acceptable to high quality predictions, however it is clear that the AlphaFold2 confidence score cannot be used to identify the best models in the set. We also show that, contrary to what was done before for CASP15‐CAPRI with AFsample, increasing the sampling without activating the dropout provides the best models for most of the targets of Round 55.

## 1 Introduction

Protein structure prediction was recently revolutionized by DeepMind's AlphaFold2 (AF2) deep learning based software [1]. At CASP14‐CAPRI in 2020, DeepMind showed predictions of monomeric structures with a quality close to the one of experimental determination [2, 3]. In 2021, they published an AlphaFold‐Multimer version adapted to protein complex prediction [4]. Version 1 came with five neural network (NN) models. However, because this release showed clashes in the prediction of some complexes, another training was performed and a new set of five NN models was released as a version 2, at the beginning of 2022. With the new CASP15‐CAPRI round happening the same year, DeepMind tried to improve again the NN parameters, resulting in a version 3 that was released the exact same day as the CASP15‐CAPRI results presentation for assembly prediction in December 2022. Thus, this last version of NN models could not be included in the assessment. However, the evaluation of the models for the assembly prediction showed that the best approaches all used AF2, in various ways [5, 6].

Many groups tweaked the alignments or refinement steps [7, 8]. Wallner's group, ranked first following CAPRI criteria [6, 9], modified the inference process. They implemented a massive sampling strategy in the AFsample tool [10], which allows to insert diversity in the inference process by tuning several parameters. These parameters are the number of predictions, the NN versions used, the number of recycling steps, the use of templates or not and the activation of the dropout at inference. The dropout is a parameter that is normally used during the training process and that allows to avoid overfitting to the training data set by randomly inactivating a percentage of the nodes of the NN. Several dropout rates were used by AF2 at many places in the process during the training and Wallner used the exact same rates for the inference. Wallner applied diverse sets of parameter combinations and predicted thousands of structures per NN, activating the dropout for all of them, at least in the Evoformer module. This approach managed, for the more difficult targets, to find one or several outliers of good quality.

In light of these results, we have developed AFmassive [11], which is an updated version of AFsample that, in particular, also includes AF2 NN v3 and allows for optional modification of the dropout rates. The primary challenge encountered when running massive sampling with AlphaFold through AFmassive or other AlphaFold‐based tools is the substantial computational time required, which can become exceedingly lengthy for demanding targets when the process is not distributed across multiple GPUs (graphics processing units). To address this challenge, we have recently developed MassiveFold [11], an optimized massive sampling tool that automatically parallelizes structure prediction computing on multiple GPUs. It allows for fast and easy computation of thousands of structures with AFmassive, ColabFold [12] and, more recently, AlphaFold3 [13].

Using MassiveFold, we applied a massive sampling strategy to the targets of CAPRI Round 55, which include three antibody–antigen complexes and a heterotrimer. We defined six sets of parameters and ran 1005 predictions per set, following primarily Wallner group's parameterization of CASP15‐CAPRI, but also including a condition with AF2 default parameters, which we call the “basic” set of parameters.

## 2 Materials & Methods

### 2.1 Targets

Protein complex predictions were run for the targets of CAPRI Round 55, which are targets T231 (antibody/peptide), T232 (serine/threonine‐protein phosphatase/TIPRL), T233 and T234 (antibody/human major histocompatibility complex‐I) (Figure S1).

### 2.2 Generation of the Predicted Structures

All predictions were obtained with MassiveFold 1.0.0 [11] on the Jean Zay supercomputing cluster at IDRIS (Institut du Développement et des Ressources en Informatique Scientifique) on V100 and A100 GPUs. Six different sets of parameters (conditions) were used combining the activation of the dropout in the Evoformer or Structure module or not and the use of templates (Table 1). One thousand five structures were generated for each set of parameters, using a different random seed for each prediction. Twenty‐one recycles and an early stop tolerance set to 0.5 (AF2 default value for early stop tolerance) were used for each condition. Thus, for each target a total of 6030 predictions were computed.

**TABLE 1:** Sets of parameters used with their respective names, 21 recycles and an early stop tolerance = 0.5 were chosen for each; dropout rates correspond to those used at training.

Setup	Dropout evoformer	Dropout structure module	Templates
Basic	No	No	Yes
WoTemplates	No	No	No
Dropout_nSM	Yes	No	Yes
Dropout_nSM_wotemplates	Yes	No	No
Dropout_full	Yes	Yes	Yes
Dropout_full_wotemplates	Yes	Yes	No

For T234, the number of acceptable models being very low (only one), 1005 extra predictions were calculated a posteriori, using the top 5 NN models of the condition with the highest ranking confidences, which was “dropout_full.” It produced one extra acceptable model. This acceptable prediction was not included in the further analyses described below but is included in the clashes section below and mentioned in the discussion.

For each target, 100 structures were submitted to CAPRI. To get some diversity in the top 5 predictions, a TM‐score was computed between the one with the highest ranking confidence and all the other predictions, and a *k*‐means clustering (*k* = 5) was calculated on these TM‐scores. The structure with the highest AF2 confidence score was taken for each cluster (top 5). The remaining 95 predictions were chosen randomly.

### 2.3 Analysis

CAPRI‐Q metrics [14] were computed for all models of the massive sampling sets and for the 100 models submitted by all the predictors, assessing each prediction against the native structure. CAPRI‐Q computes the following metrics for each prediction: the number of clashes, the fraction of native and non‐native contacts, the TM‐score, the local distance difference test (lDDT), the ligand root mean square deviation (L‐RMSD), the interface RMSD, the misorientation distance and theta angle, the sequence identity, the DockQ score [15], and the CAPRI classification. Here, we use mainly the DockQ score, which ranges from 0 to 1, where 1 corresponds to a perfect superposition of the prediction onto the native structure.

For each generated prediction, a confidence score is calculated by AF2. For multimers, this score is computed as 0.8*ipTM+0.2*pTM, where pTM is a predicted TM‐score [16] and ipTM is the pTM computed between chains [4]. The confidence scores also range from 0 to 1, with 1 being the most confident.

All plots were made in Python 3 with the seaborn 0.13.2 library [17]. 3D structure representations were rendered with Pymol 2.3.0 [18].

### 2.4 Clashes

Because the predictions were not relaxed after generation at the time of the CAPRI round 55, clashes were not taken into consideration. However, to evaluate the impact of these clashes on the ability of our massive sampling strategy to generate acceptable to high models, all acceptable or better predictions which showed an amount of clashes exceeding the CAPRI tolerance were relaxed with AMBER [19] through the colabfold_relax [12] tool, then reevaluated with the CAPRI‐Q metrics (Table 2). The relaxation results in very few predictions being rejected (< 1%, 9 instead of 1578), showing the importance of relaxation. However, we note that the overall CAPRI classification of these models is not affected; in that sense clashes have only a marginal effect on the results described in this manuscript.

**TABLE 2:** Number of predictions of acceptable or better quality that were rejected by CAPRI because of an excessive amount of clashes, before and after relaxation with AMBER.

Target	Number of rejected predictions because of excessive clashes	
	Before relaxation	After relaxation
T231	15	0
T232	1507	6
T233	42	2
T234	2	1

### 2.5 Code and Data Availability

Code used for analyses and data, which includes predicted structures and analysis results are all available here: https://gitlab.in2p3.fr/cmsb‐public/capri_55_mf.

## 3 Results and Discussion

### 3.1 Top Five Predictions Are Close to AlphaFold2 Baseline

CAPRI Round 55 included three antibody–antigen targets and a serine/threonine‐protein phosphatase‐TIPRL complex. A 3D representation of our top 1 model for each target is depicted in Figure S1 and is included for illustrative purposes in Figure 1. The best DockQ of the top five submitted predictions for all predictor and scorer groups are presented in Figure 1. The AlphaFold2 (AF2) v3 baseline is shown in blue. The three antibody–antigen targets are T231, T233, and T234, the latter two being harder to predict than T231, which shows DockQ values above 0.23 in the majority of predictions (Figure 2), roughly corresponding to at least “acceptable” solutions [15]. Our top five predictions generally perform as well as AF2 v3 with similar DockQ values, suggesting at first glance that increased sampling does not improve the quality of the predictions.

**FIGURE 1:**
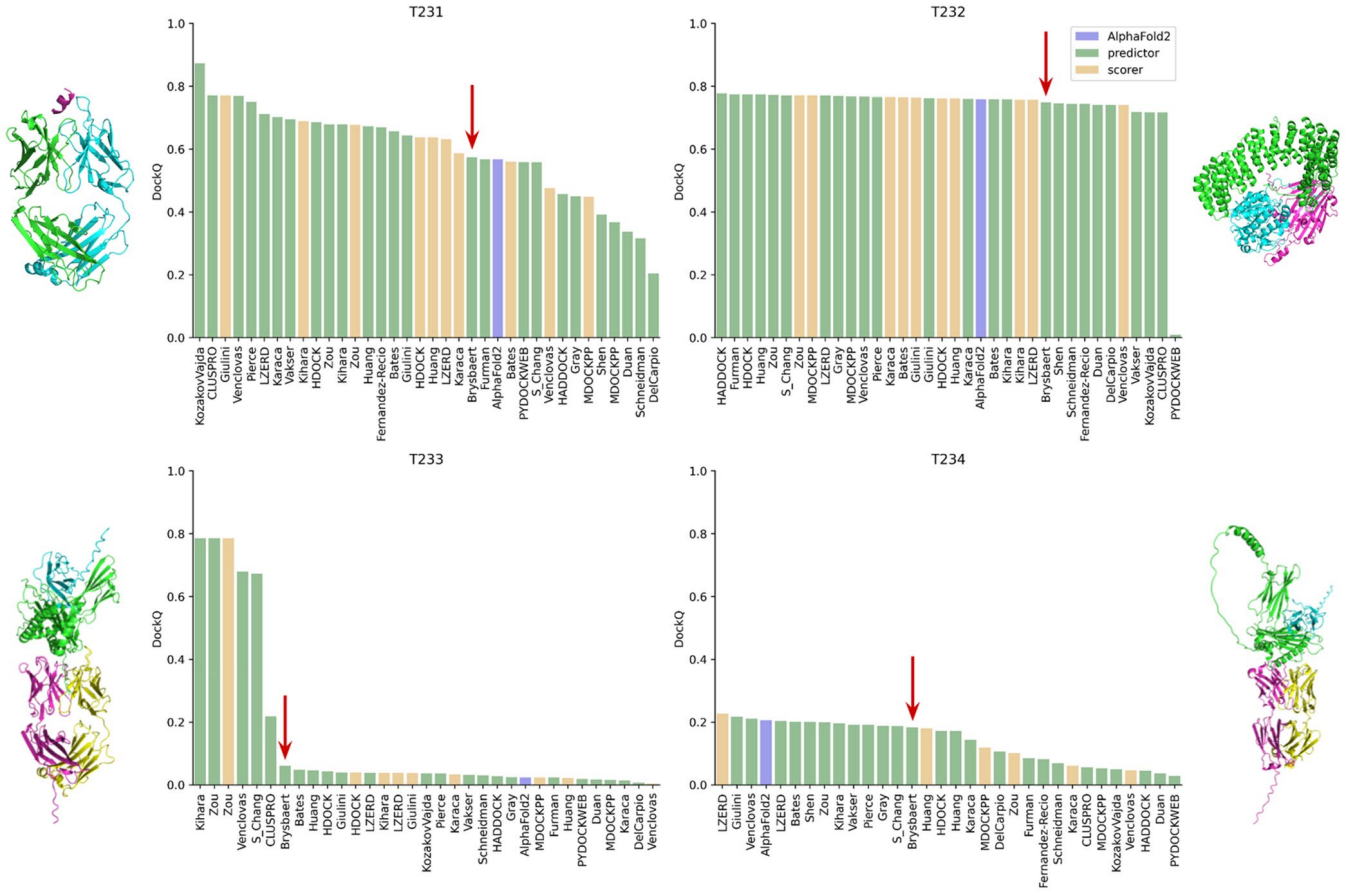
Best DockQ value in the top five submitted predictions for each predictor group (green), scorer group (yellow) and the AlphaFold2 v3 baseline (blue); a red arrow indicates our results obtained with MassiveFold; our top‐ranked structure is shown in 3D next to each target plot, each chain colored differently, as in Figure S1.

**FIGURE 2:**
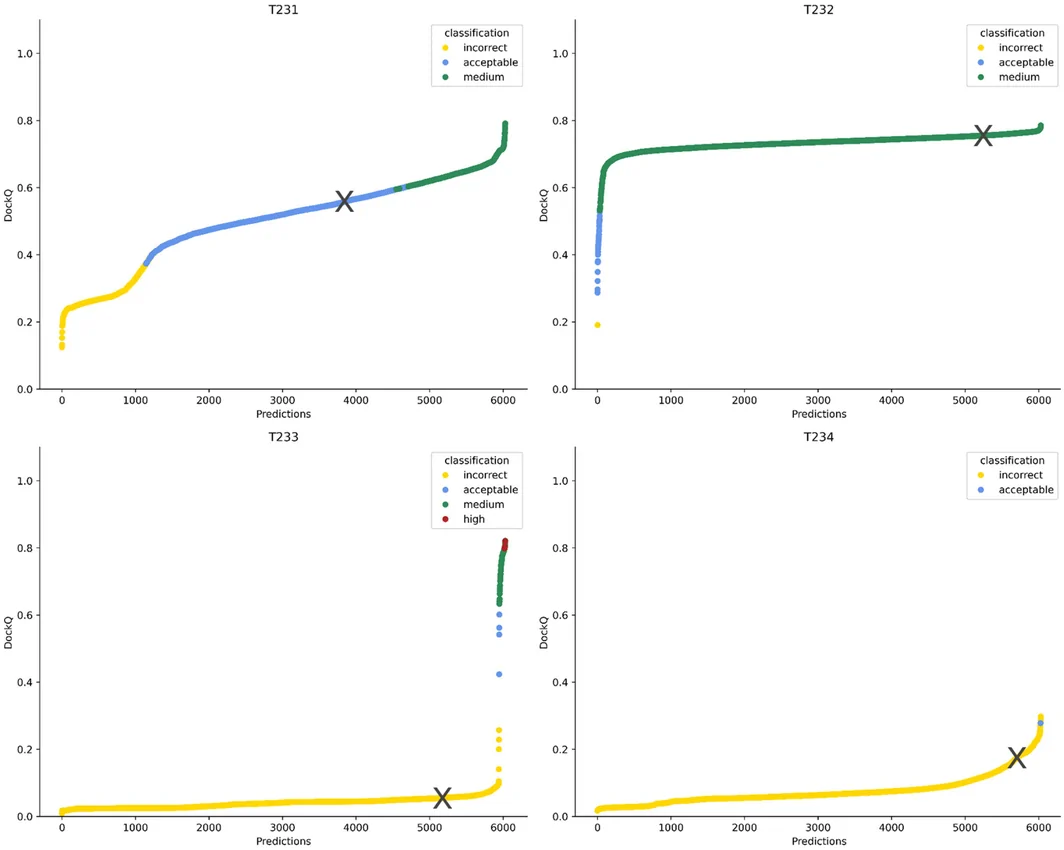
DockQ distribution for each of the four targets, ordered by ascending DockQ; the yellow/blue/green/red colors correspond to the CAPRI incorrect/acceptable/medium/high classification; the black cross indicates the model among our top five submissions with the highest DockQ.

However, Figure 2 illustrates the power of AF2 massive sampling. It shows the DockQ distribution for each target, with individual points colored following the CAPRI classification and ordered by ascending DockQ. Our best top five prediction is indicated with a black cross. This figure clearly highlights that our best top five model is not the best one in the full massive sampling set. Even, for T231 the set contains several medium quality predictions while our best top five is only of acceptable quality. For T232, the best model is of medium quality, and close to the best DockQ in the set, but here we obtained primarily medium‐quality predictions, like the other predictors and the AF2 baseline. The added value of massive sampling is more striking for T233, where our best submitted model is incorrect with a very low DockQ, whereas the full set contains models of all CAPRI categories, including four high quality predictions. The last target, T234, is also interesting because although our best submitted prediction is incorrect, we do have a single acceptable solution in the massive sampling set. Here, increased massive sampling could lead to similar behavior as observed for T233.

### 3.2 AlphaFold2 Confidence Score Fails for Antibody–Antigens Targets

These results emphasize that the AF2 confidence score, which was the central value used to select the predictions, fails to identify the best predictions in the massive sampling set for antibody–antigen targets. Figures 3 and S2 clearly show that no correlation can be observed for these targets between the AF2 ranking confidence score and the DockQ and CAPRI classification, the exception being target T232, which is not an antibody–antigen target but rather a “conventional” target. This can easily be explained by the fact that the AF2 confidence score for multimers is calculated for 80% between chains (ipTM, see method), which is heavily biased for antibody–antigen targets towards the antibody chains, with the antibody–antigen relative positions contributing less to the score. This also explains why the AF2 confidence score is *not* reliable for antibody–antigen targets but *is* reliable for nanobody‐antigen targets as shown by Wallner on the set of CASP15‐CAPRI nanobody targets [9]. A proper scoring function should be developed to take this bias into consideration and to extract the best quality models of the massive sampling set. The interface pLDDT (I‐pLDDT) as proposed by Yin and Pierce [20], which focuses on the antibody–antigen interface alone, is a promising suggestion. We computed I‐pLDDT values for all our predictions of the 3 antibody–antigen targets. While the Pearson correlation is generally poor or even negative between the DockQ and the AF2 ranking confidence (T231: −0.5/T233: 0.19/T234: −0.02), the use of I‐pLDDT improves this correlation slightly (T231: 0.14/T233: 0.19/T234: 0.24), in particular for T231, as shown in Figures S3 and S4. Indeed, Figure S3a shows that the ranking of the predictions is significantly improved for T231, when using the I‐pLDDT, as opposed to the use of AF2 ranking confidence depicted in Figure S2. Very nicely, for this target, the I‐pLDDT manages to extract a medium model as its top 1 prediction (increased DockQ shown in Figure S4). Altogether, it suggests that this metric is more adapted for peptidic antigens than for larger antigens. However, these results do show that there is still much need for improvement in the scoring of antibody–antigen targets.

**FIGURE 3:**
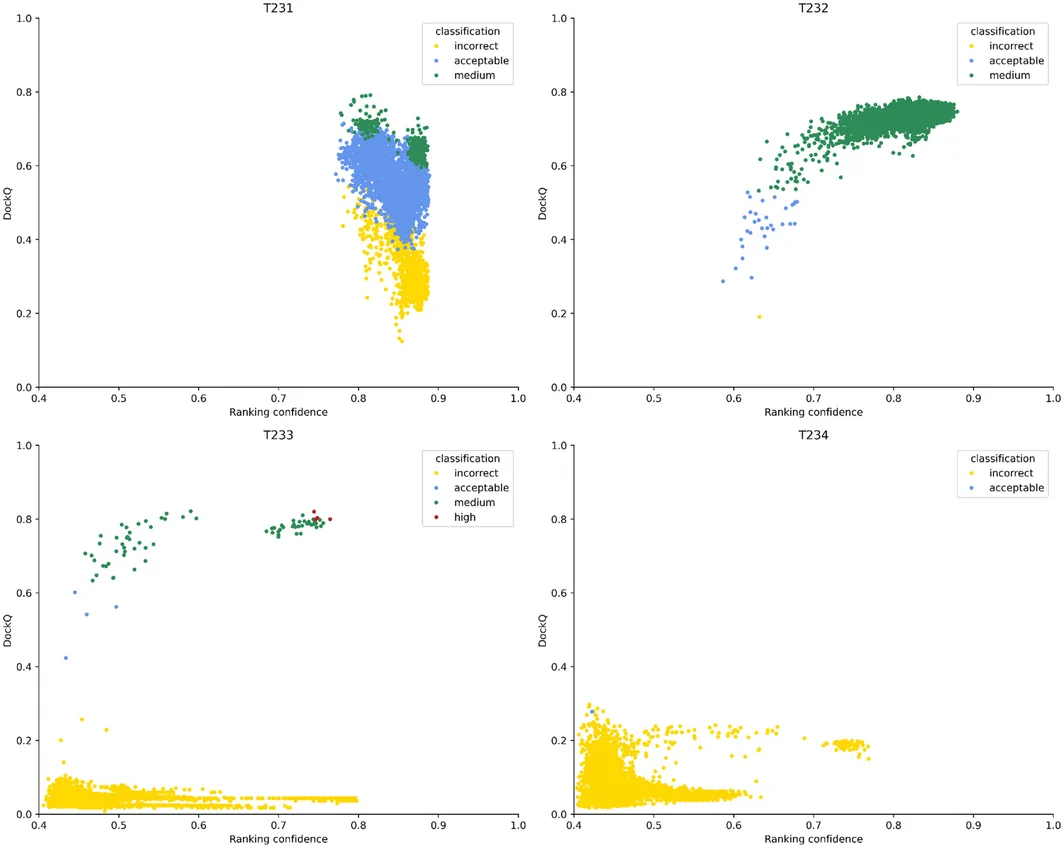
Scatter plot of the DockQ values as a function of the AlphaFold2 ranking confidence scores for the four targets of CAPRI Round 55; the yellow/blue/green/red colors correspond to the CAPRI incorrect/acceptable/medium/high classification.

### 3.3 MassiveFold Data Always Contain Predictions of Acceptable Quality or Better

If a scoring function capable of selecting the best predictions among our massive sampling sets had existed, our results would have improved significantly. To illustrate this, we computed the CAPRI metrics for all our predictions and the (up to) 100 predictions submitted by all other predictors (all available to us), and identified the highest DockQ for each group. We plotted these by descending DockQ value in Figure 4. In this case, the best prediction in the full set (MassiveFold‐best) obtains medium to high‐quality DockQ values around 0.8 for three targets including the difficult T233, and an acceptable model for the very difficult target T234. We do note that other predictors might also have such unrecognized but good quality models in their in‐house set.

**FIGURE 4:**
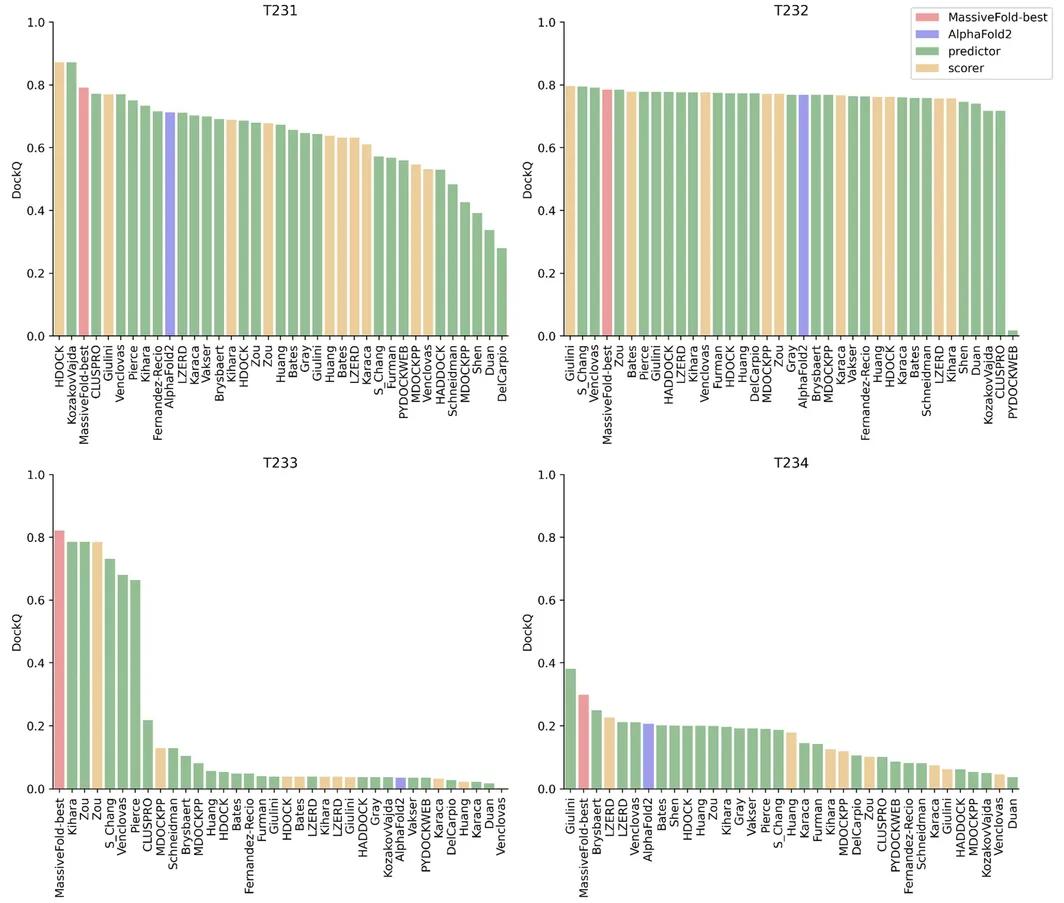
Best DockQ in the full MassiveFold dataset (red) and in all the submitted predictions for each predictor group (green), scorer group (yellow), and the AlphaFold2 v3 baseline (blue); maximum 100 predictions per target were submitted per predictor and scorer.

### 3.4 Investigating the Best Neural Network Version

Because we used three versions of AF2 NN in six different parameter sets, we attempted to identify which NN version performed best, and in particular if version 3 consistently outperformed NN versions 1 and 2, making them obsolete. Yin and Pierce [20] showed that AF2 v3 improved the success rate for antibody–antigen predictions over AF2 v2 for a set of 37 antibody–antigen complexes. In addition, the authors demonstrated on the same set that using AFsample as a massive sampling strategy increases the success rate even more. AFsample uses only AF2 NN v1 and v2. With MassiveFold, we exploit here all three AF2 NN versions. This turns out to be a good strategy because, depending on the antibody–antigen target, it is not always the same NN version that gives the best predictions. For T231, only AF2 v1 and v3 provide medium quality solutions, with v2 not resulting in better than acceptable models (Figure 5a). While all NN v1 and v3 provide medium solutions for T231, the 1st, 4th and 5th NN v1 as well as 2nd NN v3 clearly provide the highest numbers of medium quality predictions (Figure S5). For T233, only v1 and v2 provided at least acceptable solutions (Figure 6a–c), but NN v2 models were superior to v1 ones because they found the high quality structures (Figure 6c). More precisely, only the 3rd and 5th NN v2 models produced these predictions. Version 3 does not show any good prediction in this case. These two targets demonstrate that an approach, which uses all the NN models of the three versions currently available remains relevant. In this regard, for T233, the medium and high‐confidence predictions generated with v2 had more reliable AF2 confidence scores than those produced with v1, with most scores between 0.7 and 0.8, while acceptable or better predictions obtained with v1 had confidence scores below 0.6 (Figure 6c). This result has to be taken with caution because when considering only the full v2 predictions set, many high confidence scores show very low DockQ. Intriguingly, Figure S6 shows that the two NN models that produced high quality predictions are the only ones that rank well and thus manage to discriminate between good and bad predictions.

**FIGURE 5:**
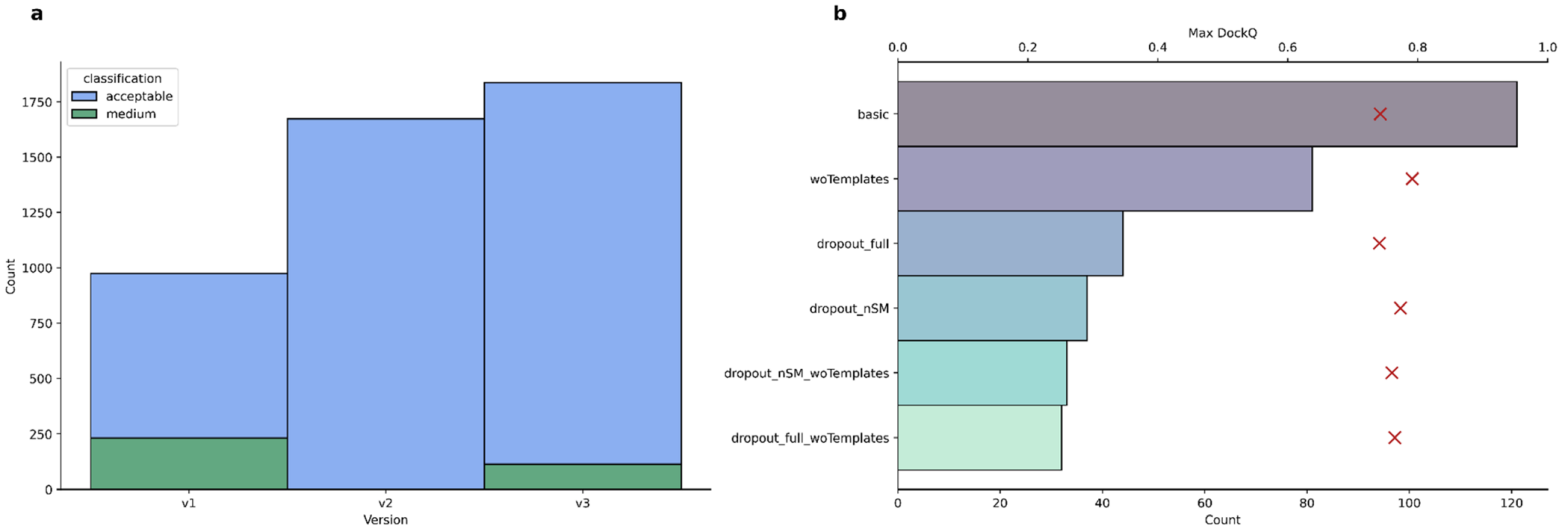
Number of predictions for T231: (a) acceptable and medium predictions per neural network versions; (b) medium quality predictions per condition (set of parameters), the bars show the number of generated models, the red crosses indicate the maximum DockQ obtained for each condition.

**FIGURE 6:**
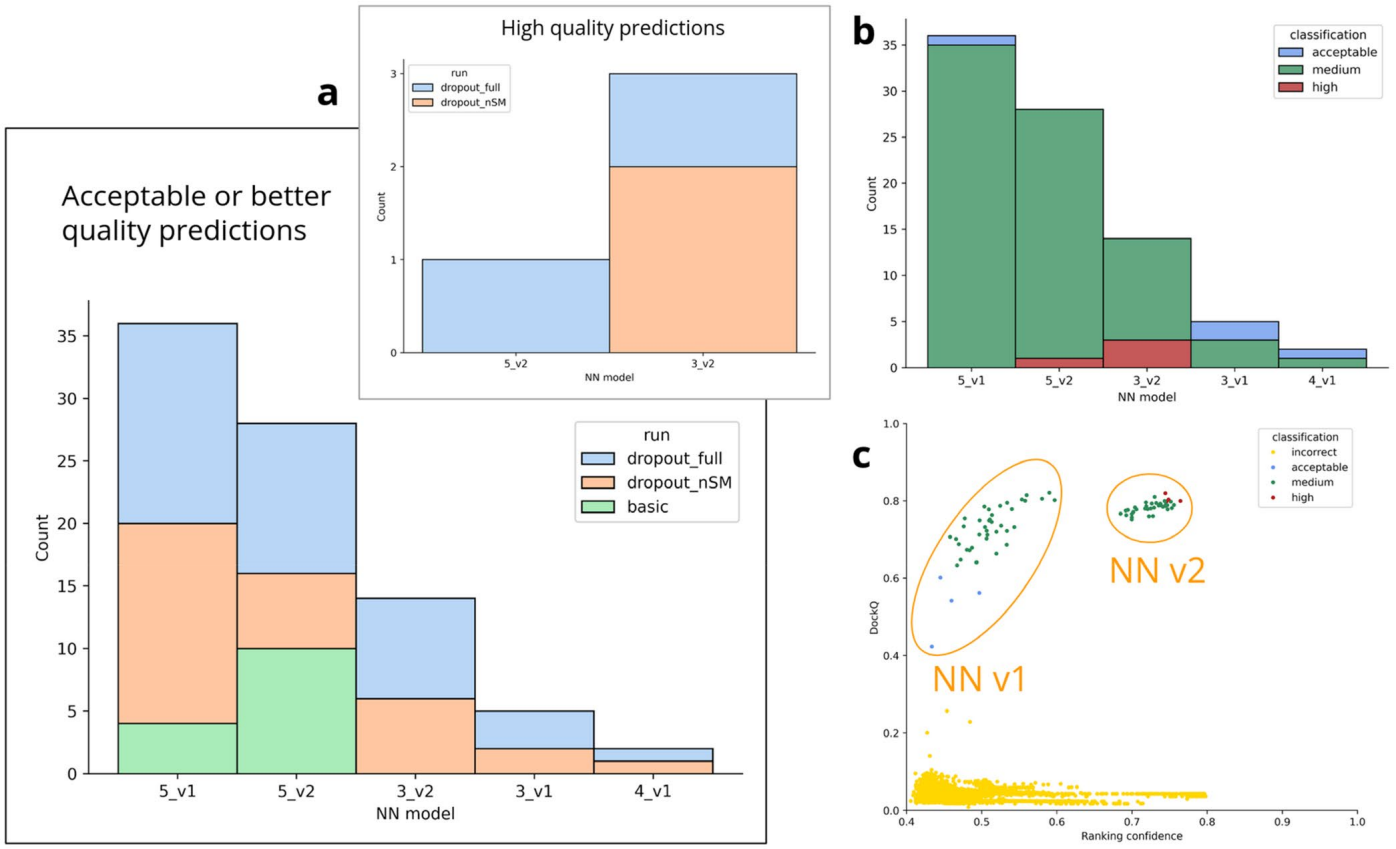
Repartition of incorrect/acceptable/medium/high quality predictions per AlphaFold2 neural network (NN) model for T233, (a) number of predictions of acceptable or better quality per NN model, the bar color indicates with which set of parameters they were found; the inset bar plot focuses on high quality predictions only; (b) repartition of acceptable, medium and high quality predictions per NN model; (c) scatter plot of DockQ as a function of ranking confidence, the yellow/blue/green/red colors correspond to the CAPRI incorrect/acceptable/medium/high classification, predictions found with AlphaFold2 NN v1 and v2 are encircled in orange; the yellow low DockQ dots stem from incorrect predictions from all three NN models.

These results on antibody–antigen targets must be put in light of AlphaFold3 (AF3), which became recently available and shows increased performance on this type of targets [13]. Focusing on the top five provided by AF3 for such targets, they are more than twice as successful as AF2 v3 (Figure 5a in [13]). Interestingly, AF3 seems even more sensitive to massive sampling, with the percentage of DockQ > 0.23 increasing significantly faster than AF2 v3 when increasing the number of seeds, although the authors only compared AF3 to AF2 v3 and not to AF2 v2 or AF2 v1. However, AF3 shows little difference in predicting antibody–antigen targets when taking only one NN diffusion sample per model seed rather than five, as performed by the AF3 server [13]. Therefore, the behavior is different between AF2 and AF3, showing the relevance of using AF3 in a massive sampling approach in addition to AF2 v1, v2, and v3. The recent publication of the AF3 code by DeepMind and our even more recent integration of this version in MassiveFold will allow this complementary approach in future massive sampling strategies.

### 3.5 Investigating the Best Set of Parameters

Next to the NN model, the set of parameters used influences the distribution of predictions. When focusing on target T231 (Figure 5b), many medium solutions were obtained with all six sets of parameters. Yet, the highest number was obtained using the “basic” set of parameters that is the default AF2 parameters. It means that only increasing the number of predictions with a different random seed and default parameters is in this case the best option to produce the maximum number of medium quality models. The “without templates” approach also yielded a high number of medium predictions, even with the highest DockQ, but, once again, dropout was not activated. The remaining parameter sets show a similar amount of medium quality predictions when the dropout in the Structure module is activated or not, suggesting a redundancy in the distribution of these sets of parameters.

For target T233, the best predictions were obtained when the dropout was activated both in the EvoFormer and the Structure module (full) or only in the EvoFormer (nSM), resulting in approximately the same amount of good predictions (Figure 6a). Here again, we observe a redundancy in the results when the dropout in the Structure module is activated or not, which can be explained by the fact that this is the lowest rate (bar zeroes) among all the dropout rates (10%). For instance, it indicates that in order to enhance diversity, one of the two conditions could be removed for another one with an increased dropout rate in the structure module. The “basic” parameterization also shows some acceptable or better predictions, highlighting again that increasing the sampling without activating the dropout does lead to good predictions. In all cases however, acceptable or better solutions were generated when using templates. T231 and T233 are two antibody–antigen targets but the first one is an antibody‐peptide target whereas the second one is an antibody‐protein target. While the optimal parameterization for T231 is using neither dropout nor templates, the parameterization of T233 is the exact contrary. And for T234 the only acceptable model was obtained with the “basic” parameterization. Unfortunately, this model contained many clashes, which an additional refinement step did not resolve. Consequently, for this target, an extra set of 1005 predictions was calculated using the top five NN models, which gave the highest ranking confidences for the “dropout_full” condition. It produced one additional acceptable solution, which showed a tolerated amount of clashes after relaxation. Since this was an exceptionally difficult target, increasing even more the number of predictions may lead to better results. Although the target set of CAPRI Round 55 is small, the results clearly show the promise of massive sampling with multiple parameter sets, and in particular with the “basic,” default parameters.

The number of predictions per parameter set is also an aspect one can play with. Figure S7 shows that the minimum number of predictions needed to reach the maximum DockQ value varies for each target, going from 10 predictions per NN model for T234 (basic) to about 50 for T232 (basic), suggesting no predefined ruleset for the number of predictions to calculate. For T233, “dropout_full” and “dropout_nSM” directly produce a very good first structure with a DockQ around 0.8, demonstrating that for this target the variability in the parameters is significantly more important than increasing the number of predictions. Incidentally, the plot again shows that the “dropout_full” and “dropout_nSM” conditions look redundant, irrespective of template use, and, above all, that the highest DockQ values are obtained for three targets out of four without activating the dropout (“basic” or “woTemplates”), with the remaining one, T233, showing a DockQ > 0.75 for the “basic” set of parameters, albeit not the highest one obtained. The difficulty for these antibody–antigen targets lies in the unreliability of the AF2 confidence score. If a more trustable score was designed, a time and power saving approach would be to start from the default five predictions per NN model used by AF2 and compute iteratively an extra five (or more) per NN model if a defined threshold is not reached by the highest score. Consequently, using four sets of parameters (with optionally the extra 2 “dropout_nSM” ones) seems relevant in a general usage and in the antibody–antigen case in particular.

MassiveFold was recently used in CASP16‐CAPRI to generate a massive sampling baseline and the produced data were provided to all modelers for the stage 2 prediction round, where they could use them in any way to provide their new top 5 predictions, additionally to being included in the CAPRI scoring round. For this experiment, we chose eight conditions, replacing one nSM condition with a condition with three recycles, and added two extra conditions where ColabFold was used; since v1.2, MassiveFold integrates ColabFold, which is based on the MMseqs2 alignment tool run on the ColabFold database. CASP16‐CAPRI is a massive application of MassiveFold and is expected to teach us which set of parameters works best on which types of targets, including monomers, multimers, and the specific case of antibody‐antigens.

## 4 Conclusion

We adopted a massive sampling strategy to model protein–protein and protein‐peptide complexes in CAPRI Round 55. The strategy had already shown its worth in CASP15‐CAPRI and was extended here. However, the specific case of antibody–antigen targets remains difficult. Our results show that although the AlphaFold2 confidence score performs generally well to rank the predictions for “conventional” targets like the heterotrimeric target T232, it completely fails to rank the best predictions for the antibody–antigen targets. Nonetheless, they also show that even if our top 5 structures are not always of acceptable quality (or better), our prediction pool always contains acceptable to high quality predictions. The challenge remains to score the predictions properly to be able to identify them as near native. Using various sets of parameters and all the AlphaFold2 neural network versions in a tool like MassiveFold provides distributions of predictions and increases the likelihood of obtaining a good one, but very good results can already be achieved simply by increasing the number of predictions with AlphaFold2 default parameters. Even better predictions could be obtained by exploring additional tools such as ColabFold or AlphaFold3, which have by now been integrated into MassiveFold.

## Author Contributions

**Nessim Raouraoua:** conceptualization, investigation, methodology, writing – original draft, validation, visualization, software, formal analysis, data curation, resources. **Marc F. Lensink:** conceptualization, writing – original draft, validation, writing – review and editing, project administration. **Guillaume Brysbaert:** conceptualization, investigation, funding acquisition, writing – original draft, methodology, validation, writing – review and editing, software, formal analysis, project administration, supervision, resources.

## Conflicts of Interest

The authors declare no conflicts of interest.

## Supporting information


Data S1.

## Data Availability

Code used for analyses and data, which includes predicted structures and analysis results are all available here: https://gitlab.in2p3.fr/cmsb‐public/capri_55_mf.

## References

[1] J. Jumper , R. Evans , A. Pritzel , et al., “Highly Accurate Protein Structure Prediction With AlphaFold,” Nature 596, no. 7873 (August 2021): 583–589. 10.1038/s41586-021-03819-2 PMC8371605 34265844

[2] M. F. Lensink , G. Brysbaert , T. Mauri , et al., “Prediction of Protein Assemblies, the Next Frontier: The CASP14‐CAPRI Experiment,” Proteins 89, no. 12 (Decemeber 2021): 1800–1823. 10.1002/prot.26222 PMC8616814 34453465

[3] A. Kryshtafovych , T. Schwede , M. Topf , K. Fidelis , and J. Moult , “Critical Assessment of Methods of Protein Structure Prediction (CASP)‐round XIV,” Proteins 89, no. 12 (Decemeber 2021): 1607–1617. 10.1002/prot.26237 PMC8726744 34533838

[4] R. Evans , M. O'Neill , A. Pritzel , et al., “Protein Complex Prediction With AlphaFold‐Multimer,” bioRxiv (2021), 10.1101/2021.10.04.463034v1.

[5] A. Kryshtafovych , T. Schwede , M. Topf , K. Fidelis , and J. Moult , “Critical Assessment of Methods of Protein Structure Prediction (CASP)‐round XV,” Proteins 91, no. 12 (Decemeber 2023): 1539–1549. 10.1002/prot.26617 PMC10843301 37920879

[6] M. F. Lensink , G. Brysbaert , N. Raouraoua , et al., “Impact of AlphaFold on Structure Prediction of Protein Complexes: The CASP15‐CAPRI Experiment,” Proteins 91, no. 12 (Decemeber 2023): 1658–1683. 10.1002/prot.26609 PMC10841881 37905971

[7] T. Oda , “Improving Protein Structure Prediction With Extended Sequence Similarity Searches and Deep‐Learning‐Based Refinement in CASP15,” Proteins 91, no. 12 (December 2023): 1712–1723. 10.1002/prot.26551 37485822

[8] W. Zheng , Q. Wuyun , Y. Li , C. Zhang , P. L. Freddolino , and Y. Zhang , “Improving Deep Learning Protein Monomer and Complex Structure Prediction Using DeepMSA2 With Huge Metagenomics Data,” Nature Methods 21, no. 2 (February 2024): 279–289. 10.1038/s41592-023-02130-4 PMC10864179 38167654

[9] B. Wallner , “Improved Multimer Prediction Using Massive Sampling With AlphaFold in CASP15,” Proteins 91 (August 2023): 1734–1746. 10.1002/prot.26562 37548092

[10] B. Wallner , “AFsample: Improving Multimer Prediction With AlphaFold Using Massive Sampling,” Bioinformatics 39, no. 9 (September 2023): btad573. 10.1093/bioinformatics/btad573 PMC10534052 37713472

[11] N. Raouraoua , C. Mirabello , T. Véry , et al., “MassiveFold: Unveiling AlphaFold's Hidden Potential With Optimized and Parallelized Massive Sampling,” Nature Computational Science 4, no. 11 (2024): 824–828. 10.1038/s43588-024-00714-4 PMC11578886 39528570

[12] M. Mirdita , K. Schütze , Y. Moriwaki , L. Heo , S. Ovchinnikov , and M. Steinegger , “ColabFold: Making Protein Folding Accessible to all,” Nature Methods 19, no. 6 (2022): 679–682. 10.1038/s41592-022-01488-1 PMC9184281 35637307

[13] J. Abramson , J. Adler , J. Dunger , et al., “Accurate Structure Prediction of Biomolecular Interactions With AlphaFold 3,” Nature 630, no. 8016 (June 2024): 493–500. 10.1038/s41586-024-07487-w PMC11168924 38718835

[14] K. W. Collins , M. M. Copeland , G. Brysbaert , et al., “CAPRI‐Q: The CAPRI Resource Evaluating the Quality of Predicted Structures of Protein Complexes,” Journal of Molecular Biology 436, no. 17 (March 2024): 168540. 10.1016/j.jmb.2024.168540 PMC11458157 39237205

[15] S. Basu and B. Wallner , “DockQ: A Quality Measure for Protein‐Protein Docking Models,” PLoS One 11, no. 8 (August 2016): e0161879. 10.1371/journal.pone.0161879 PMC4999177 27560519

[16] Y. Zhang and J. Skolnick , “Scoring Function for Automated Assessment of Protein Structure Template Quality,” Proteins: Structure, Function, and Bioinformatics 57, no. 4 (2004): 702–710. 10.1002/prot.20264 15476259

[17] M. L. Waskom , “Seaborn: Statistical Data Visualization,” Journal of Open Source Software 6, no. 60 (April 2021): 3021.

[18] L. L. C. Schrödinger , “The PyMOL Molecular Graphics System,” Version 1 (2015): 8.

[19] V. Hornak , R. Abel , A. Okur , B. Strockbine , A. Roitberg , and C. Simmerling , “Comparison of Multiple Amber Force Fields and Development of Improved Protein Backbone Parameters,” Proteins 65, no. 3 (2006): 712–725. 10.1002/prot.21123 PMC4805110 16981200

[20] R. Yin and B. G. Pierce , “Evaluation of AlphaFold Antibody‐Antigen Modeling With Implications for Improving Predictive Accuracy,” Protein Science 33, no. 1 (January 2024): e4865. 10.1002/pro.4865 PMC10751731 38073135

