# Supplementary material for: Massive Sampling Strategy for Antibody–Antigen Targets in CAPRI Round 55 With MassiveFold
Source: Proteins. 2025 Jan 27;94(11):1768–76. doi: 10.1002/prot.26802 (PMC13648903; doi:10.1002/prot.26802)
Supplement: Supplementary file 1 [file PROT-94-1768-s001.pdf]

Massive sampling strategy for antibody-antigen targets in CAPRI Round 55 with MassiveFold

Supplementary Material

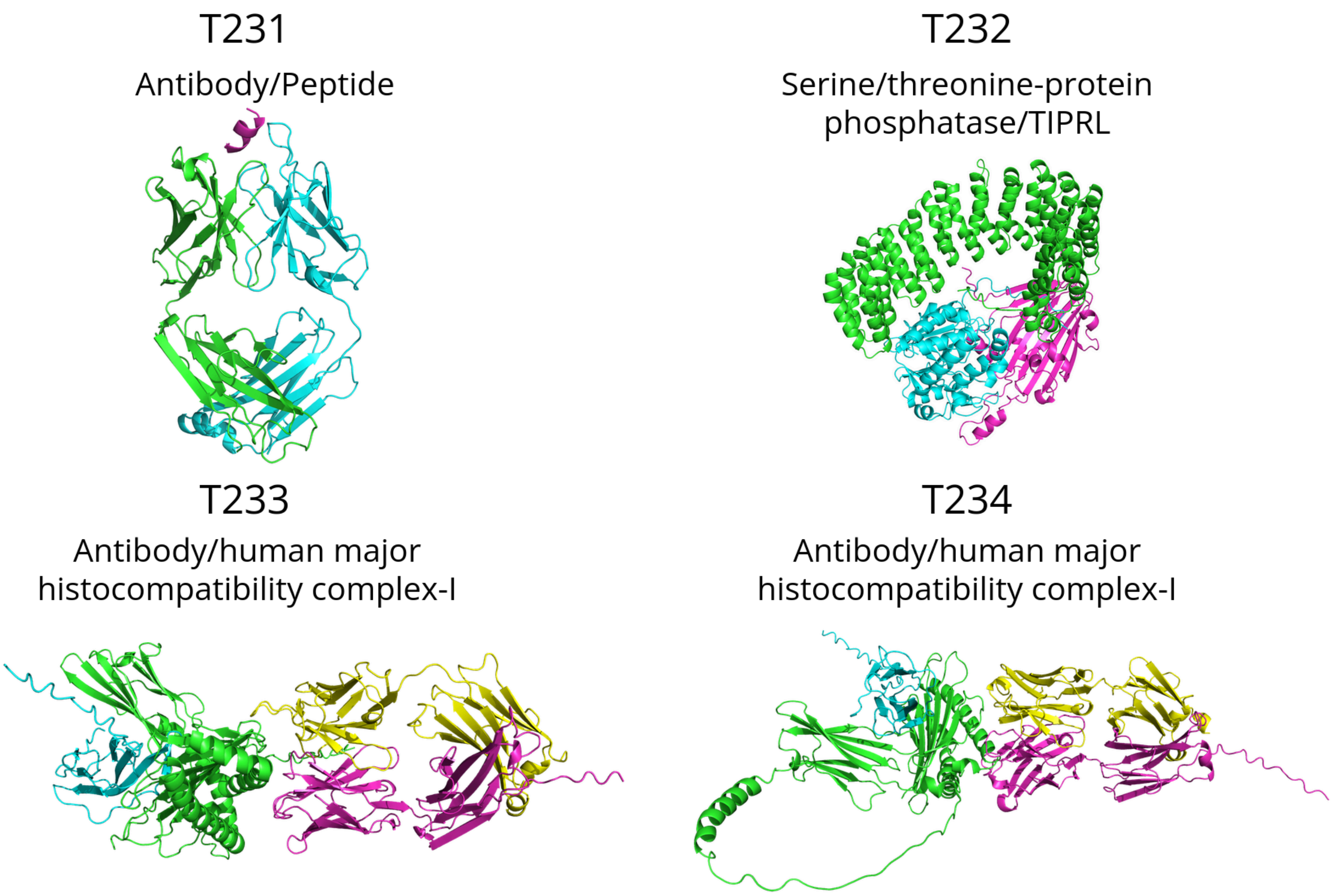

Figure S1: 3D representation of our top ranked prediction generated with MassiveFold, for each of the targets in CAPRI Round 55; the top ranked prediction was selected following the AlphaFold ranking confidence score and does not correspond to the best prediction in the set; each chain is colored differently to distinguish them.

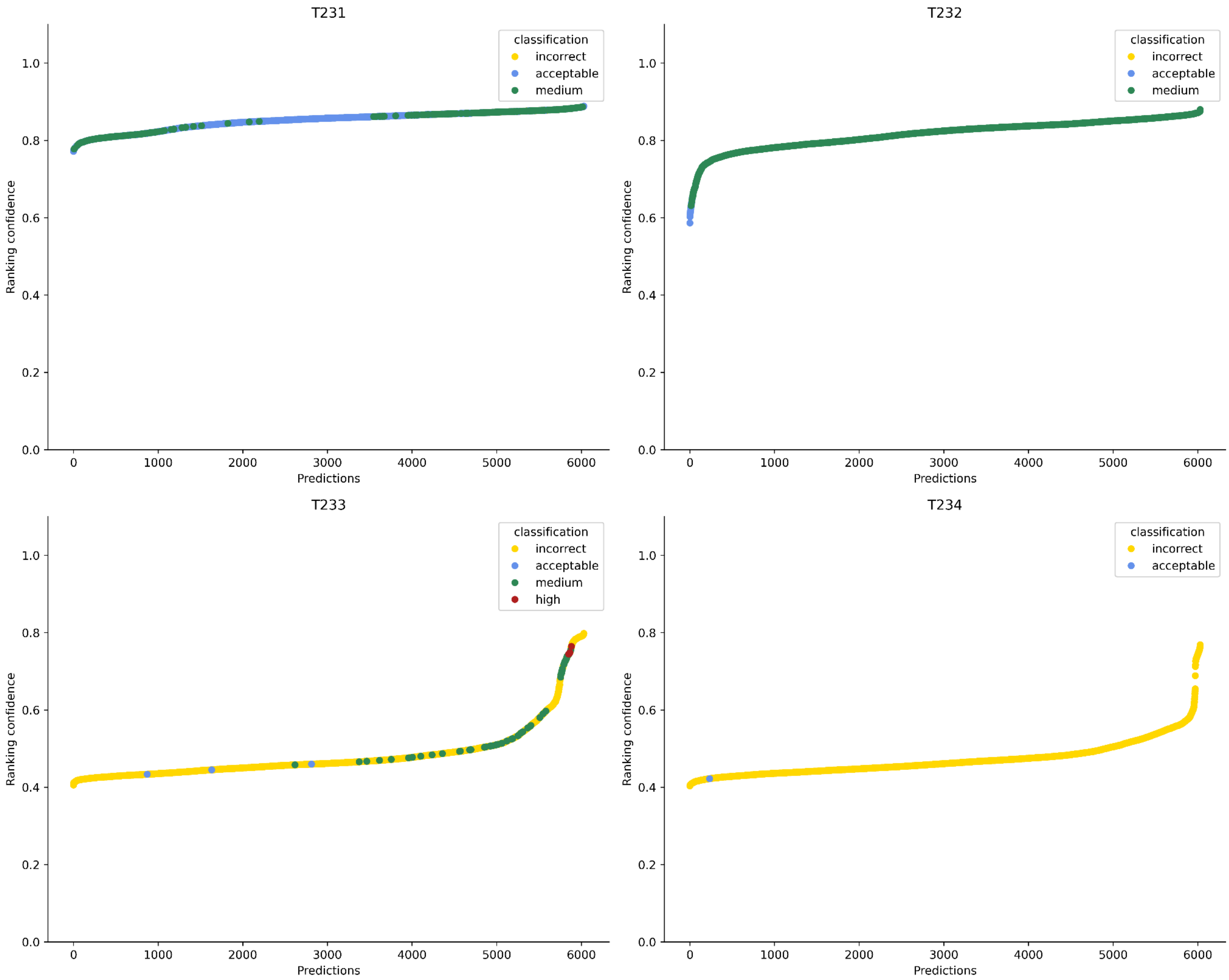

Figure S2: AlphaFold ranking confidence for each prediction of each of the targets of CAPRI Round 55, ordered by ascending ranking confidence; the yellow/blue/green/red colors correspond to the CAPRI incorrect/acceptable/medium/high classification.

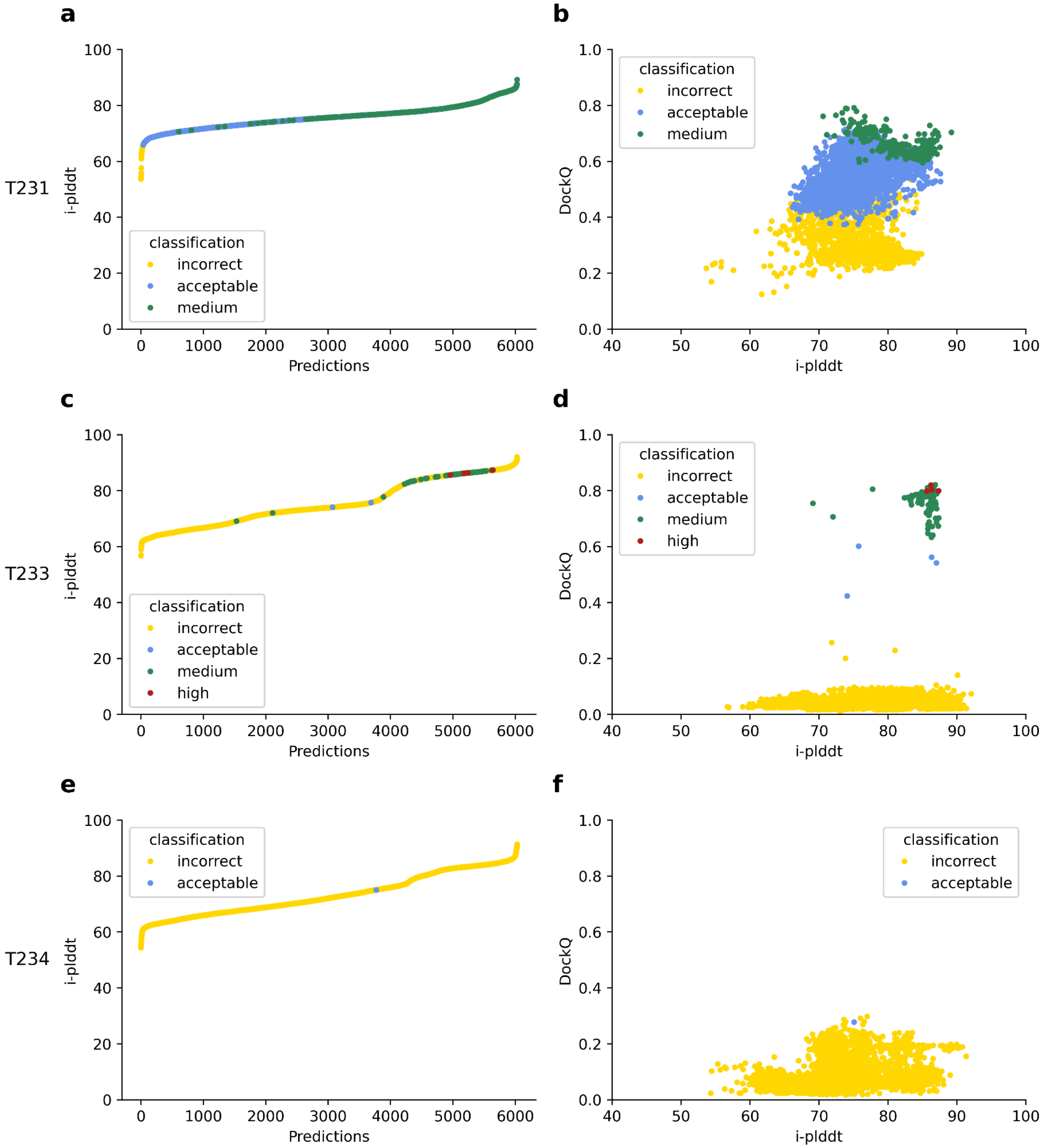

Figure S3: I-pLDDT scoring. (**ace**) I-pLLDT for each prediction of each of the antibody-antigen targets of CAPRI Round 55, ordered by ascending I-pLDDT; the yellow/blue/green/red colors correspond to the CAPRI incorrect/acceptable/medium/high classification; (**bdf**) scatter plot of the DockQ values in function of the I-pLDDT scores.

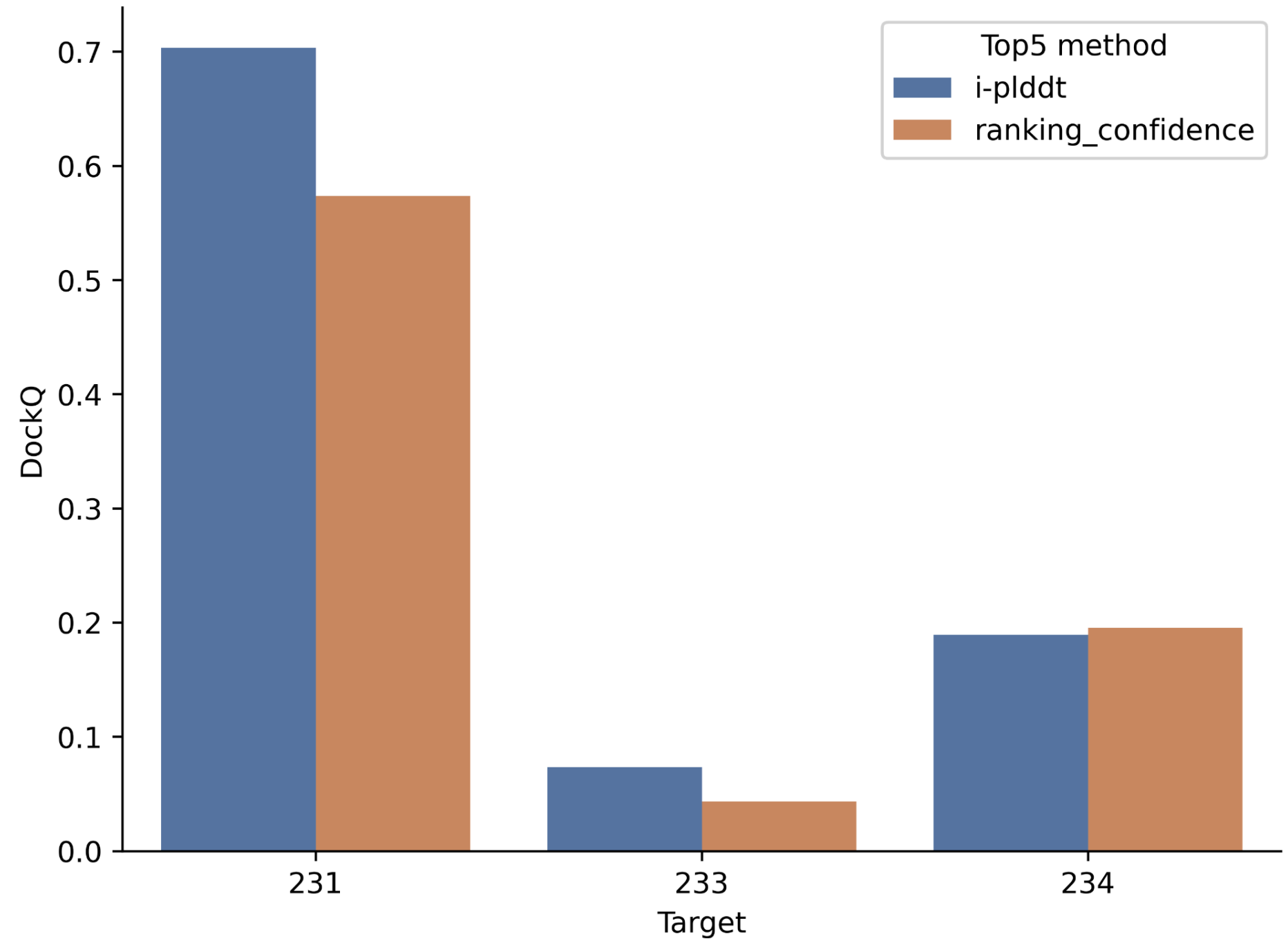

Figure S4: Comparison between the maximum DockQ of the top 5 predictions for the three antibody-antigen targets.

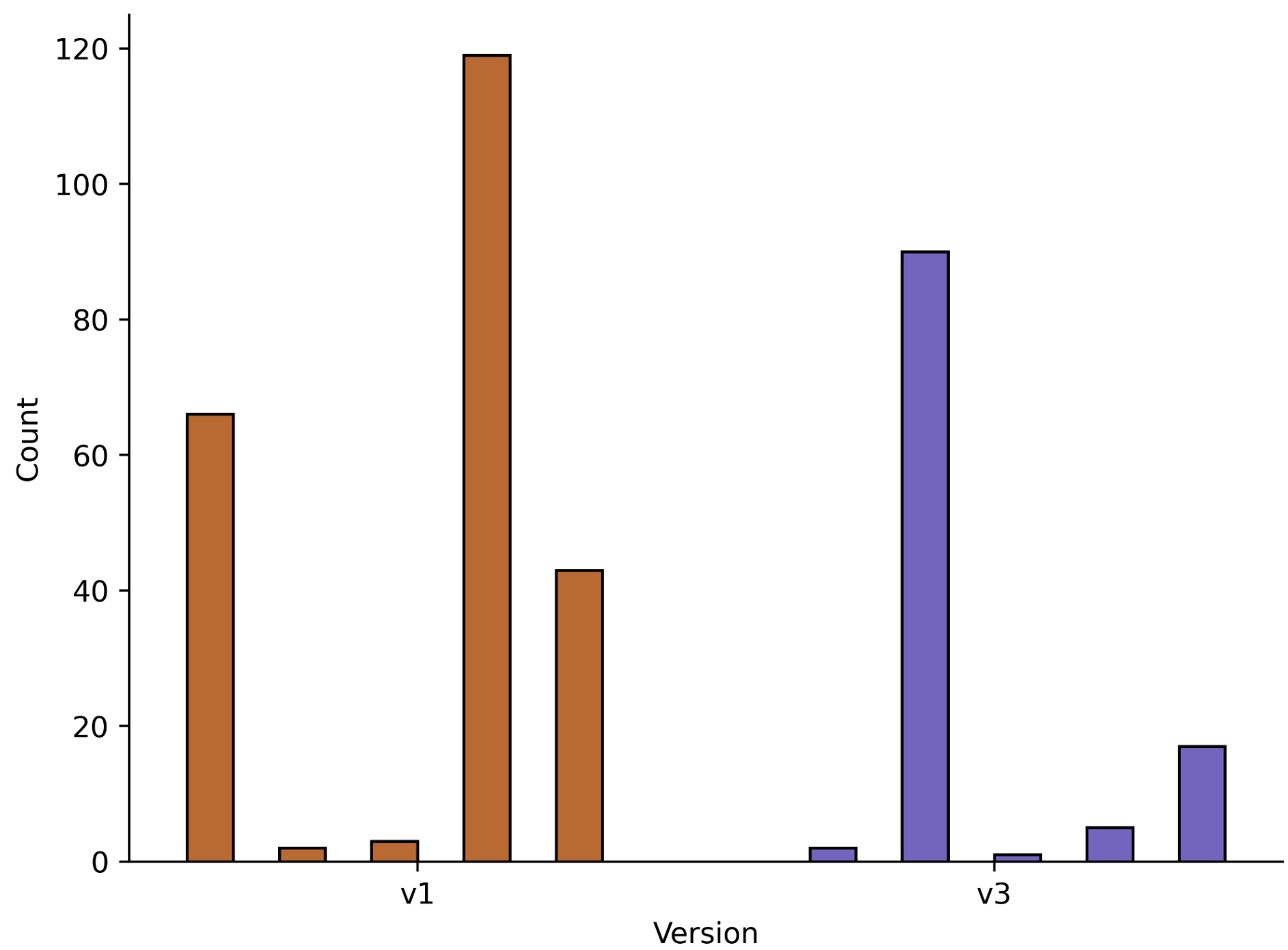

Figure S5: Number of medium predictions per neural network (NN) model for **T231**; the neural networks are ordered by version number; AF2 NN v2 is not present because no medium quality predictions were generated by these NN models.

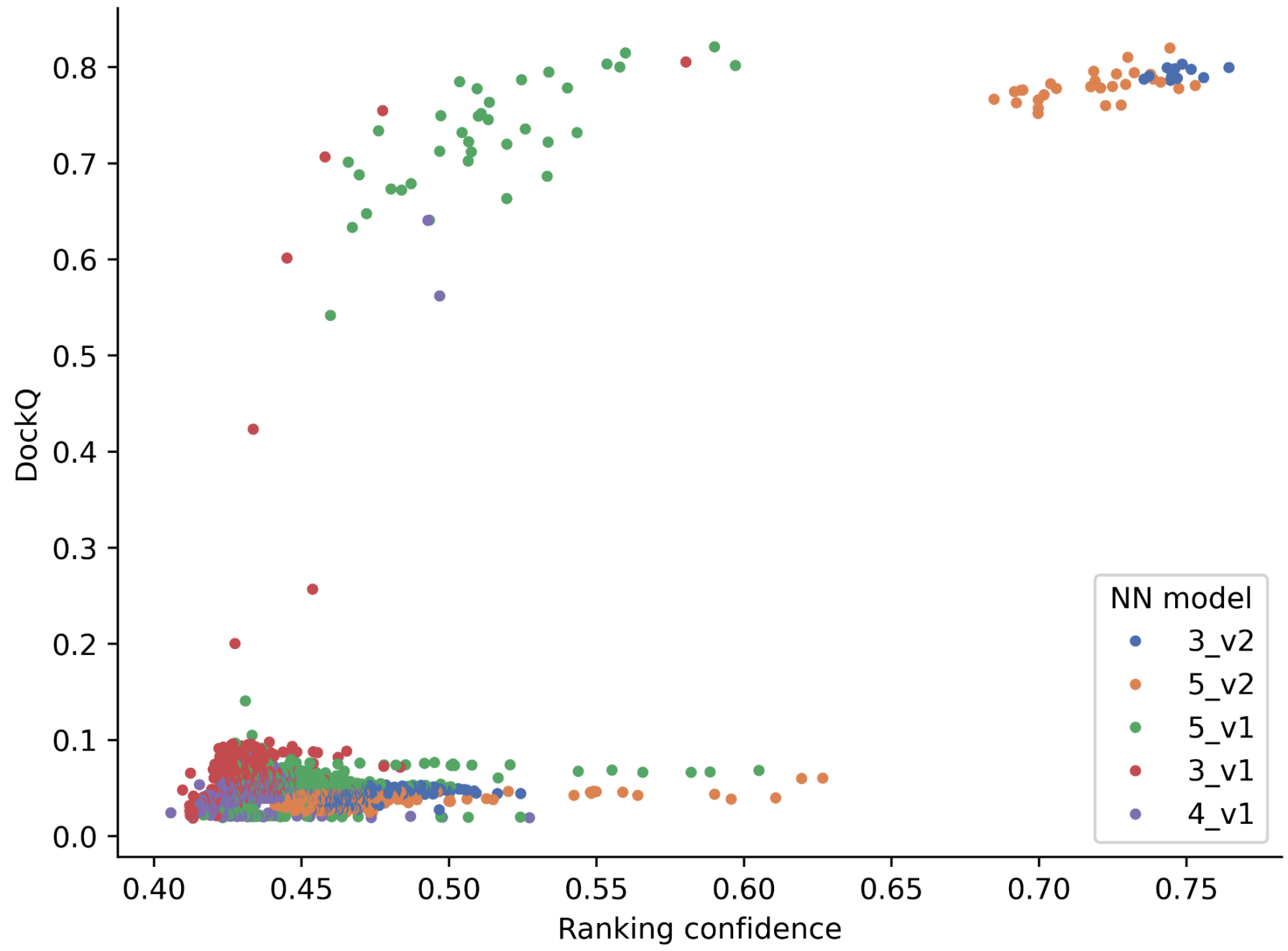

Figure S6: DockQ *vs.* AlphaFold ranking confidence, for **T233**; only the 5 neural network (NN) models that produced at least acceptable predictions are plotted, but all the predictions for these 5 are shown.

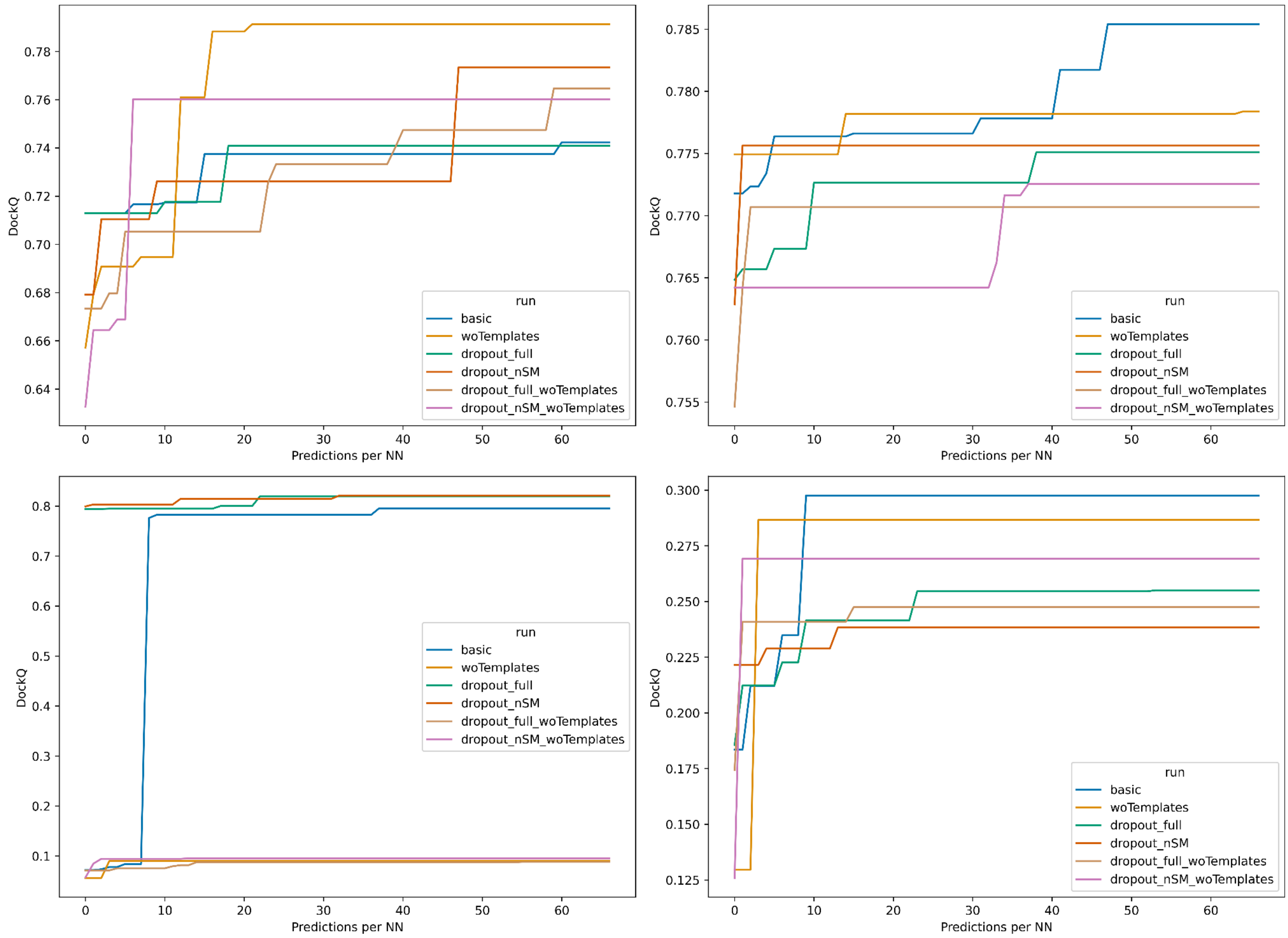

Figure S7: Evolution of the maximum DockQ in function of the number of predictions per neural network (NN) model for **T231** (top left), **T232** (top right), **T233** (bottom left) and **T234** (bottom right).
